# Supplementary material for: Modeling the effectiveness of olfactory testing to limit SARS-CoV-2 transmission
Source: Nat Commun. 2021 Jun 16;12:3664. doi: 10.1038/s41467-021-23315-5 (PMC8209051; doi:10.1038/s41467-021-23315-5)
Supplement: Supplementary file 1 — Supplementary Information [file 41467_2021_23315_MOESM1_ESM.pdf]

Modeling the effectiveness of olfactory testing to limit SARS-CoV-2 transmission

Supplementary Figures and Tables

Daniel B. Larremore, Derek Toomre, and Roy Parker

daniel.larremore@colorado.edu

Contents

- Supplementary Figures 1-5
- Supplementary Table 1
- Supplementary Note

Reference Author	Objective Olfactory Test Type	Country	N	Olfactory Dysfunction[b]	Comments
Moein et al. [1]	UPSIT (40 odorants)	Iran	60	98% [91,100]	Adults, moderate and severe
Vaira et al. [2]	CCCRC	Italy	72	61% [65,74]	Adults, symptomatic
Hornuss et al. [3]	Sniffin' Sticks	Germany	45	84% [71,92]	Adults, symptomatic
Lechien et al. [4]	Sniffin' Sticks	Belgium	86	84% [35,67]	Adults, symptomatic
Vaira et al. [5]	CCCRC	Italy	33	52% [35,67]	Adults, symptomatic
Vaira et al. [6]	CCCRC	Italy	345	70% [65,74]	Adults, symptomatic
Bhattacharjee et al. [7]	Custom	India	70[a]	82% [N.D.]	Adults, asymptomatic. 15% self-reported LOS
Rusetsky et al. [8]	Sniffin' Sticks SIT	Russia	79	86% [N.D.]	Children. 48% recovered from LOS after 5 days.

Supplementary Table 1: **Frequency of olfactory dysfunction (OD) in COVID-19 subjects when monitored with a quantitative olfactory test device.** For all studies, PCR for SARS CoV-2 was used as the "gold standard" reference test. Data are expanded and modified from [9] and [10]. SIT, Smell Identification Test; CCRC, Connecticut Chemosensory Clinical Research Center; LOS, Loss of Smell; N.D., not determined.

[a] 37 healthy and 33 asymptomatic COVID-19 positive patients.

[b] Percentages represent central estimates, with 95% confidence intervals in brackets. Across studies, most tests were performed a single point in the clinical course. Percentage of PCR-positive COVID-19 cases with confirmed OD are therefore estimates of the sensitivity of OD as a predictor of OD. True prevalence of the symptom may therefore vary if windows of PCR positive and OD are imperfectly overlapping.

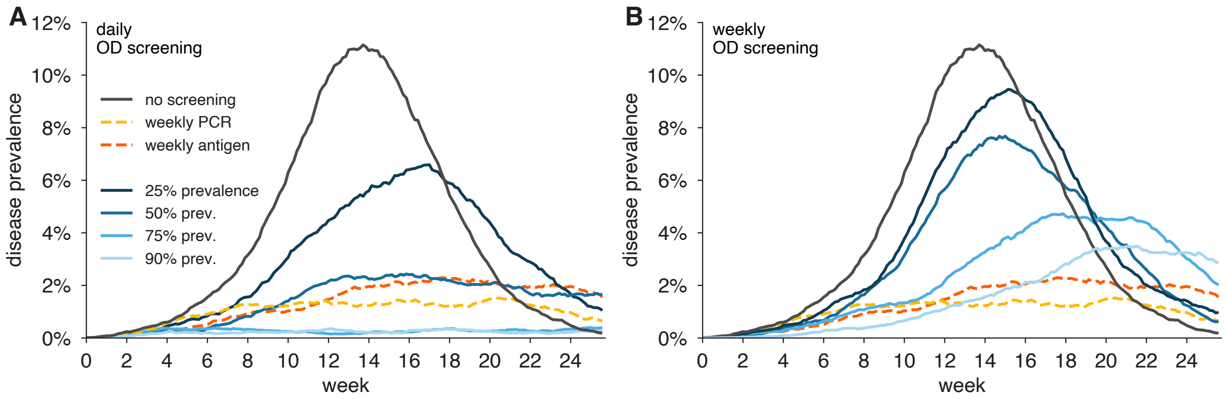

Supplementary Figure 1: **Impact of olfactory dysfunction prevalence on its effectiveness to limit viral spread.** The figure shows examples of viral spread in fully-mixed community of 20,000 individuals performing olfactory dysfunction (OD) screening daily (A), or weekly (B). No mitigation (black), Prevalence of symptom shown are: 25% (dark blue), 50% (medium blue), 75% (light blue), 90% (lightest blue). For comparison, weekly RT-PCR testing with a one day turnaround is shown. In this analysis, olfactory dysfunction is modeled to last 7 days, and begin two days after viral levels reach 1000 virions/ml. We consider 80% participation in testing and that 20% of individuals would suffer COVID-19-independent olfactory dysfunction which would exclude them from effective testing.

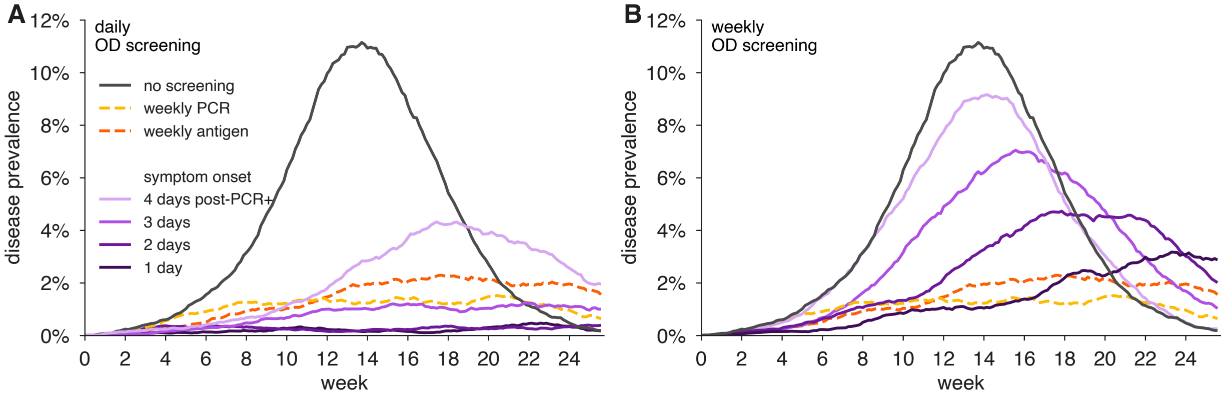

Supplementary Figure 2: **Impact of the timing of olfactory dysfunction onset on its effectiveness to limit viral spread.** Example of viral spread in fully-mixed community of 20,000 individuals performing olfactory dysfunction (OD) screening daily (A) or weekly (B). olfactory dysfunction is modeled to be present in 75% of infected individuals, and to last 7 days. We consider 80% participation in testing and that 20% of individuals would suffer COVID-19-independent olfactory dysfunction which would exclude them from effective testing. Timing of olfactory dysfunction is varied from one to four days after virions levels reaching 1000 virions/ml (purple shaded lines as indicated). No mitigation is shown as black line. For comparison, weekly RT-PCR testing with a one day turnaround is shown.

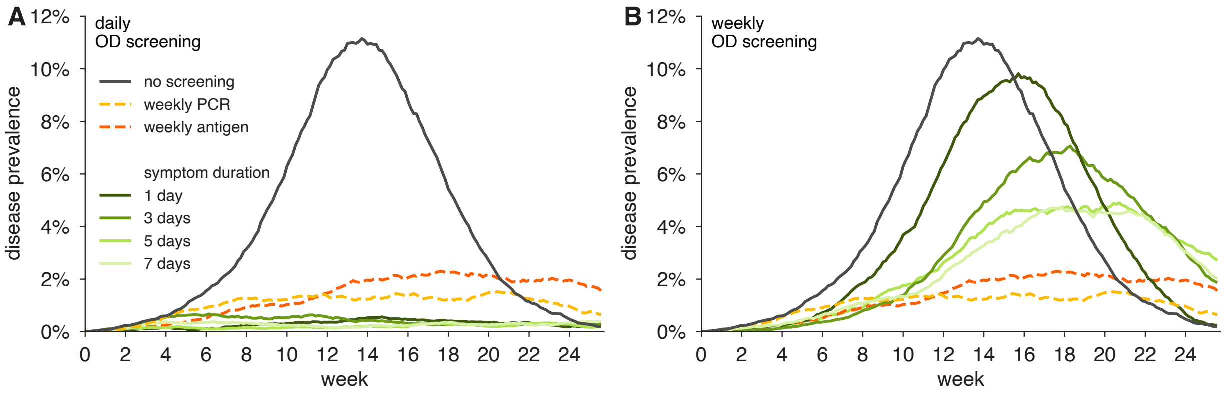

Supplementary Figure 3: **Impact of duration of olfactory dysfunction on its effectiveness to limit viral spread.** Examples of viral spread in fully-mixed community of 20,000 individuals performing olfactory dysfunction (OD) screening daily (A) or weekly (B). Olfactory dysfunction is modeled to be present in 75% of infected individuals, and to begin two days after virion levels reach 1000 virions/ml. We consider 80% participation in testing and that 20% of individuals would suffer COVID-19-independent olfactory dysfunction which would exclude them from effective testing. Duration of olfactory dysfunction is varied from 7 days (lightest green), 5 days (light green), 3 days (green), to 1 day (dark green). No mitigation is shown as black line. For comparison, weekly RT-PCR testing with a one day turnaround is shown.

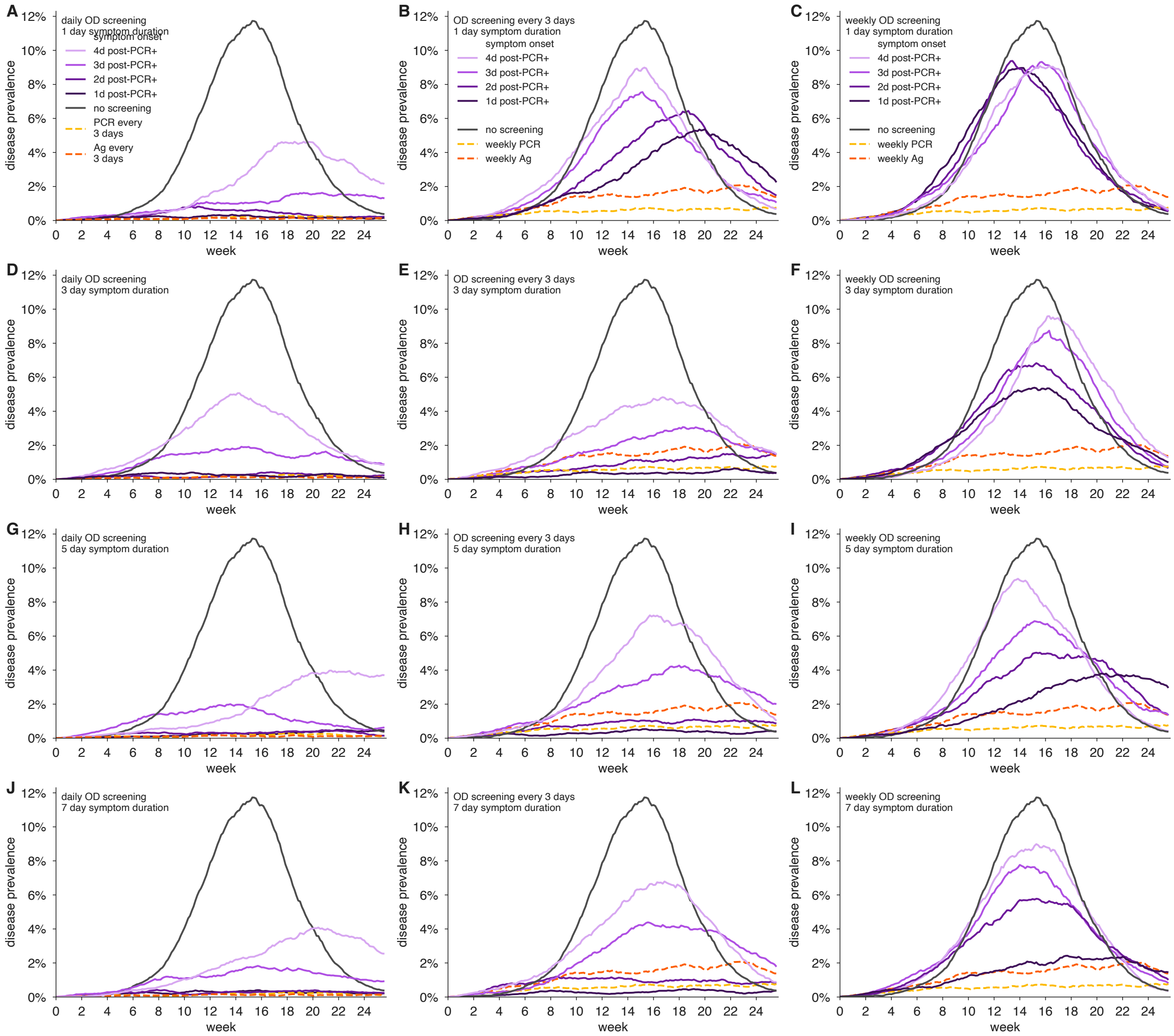

Supplementary Figure 4: **Impact of varying duration and onset time of olfactory dysfunction on its effectiveness to limit viral spread.** Examples of viral spread in fully-mixed community of 20,000 individuals performing olfactory dysfunction (OD) screening daily for symptoms lasting (A, B, C) one day, (D, E, F) three days, (G, H, I) five days, and (J, K, L) seven days, screening every day (left column), every three days (middle column) and weekly (right column). olfactory dysfunction is modeled to be present in 75% of infected individuals, and to begin after 1, 2, 3, or 4 days from when virion levels reach 1000 virions/ml, indicated by varying shades of purple (see legend). We consider 80% participation in testing and that 20% of individuals would suffer COVID-19-independent olfactory dysfunction which would exclude them from effective testing. No mitigation is shown as black line. For comparison, RT-PCR testing with a one day turnaround and antigen testing are shown with testing every three days (left column) or weekly (middle and right columns).

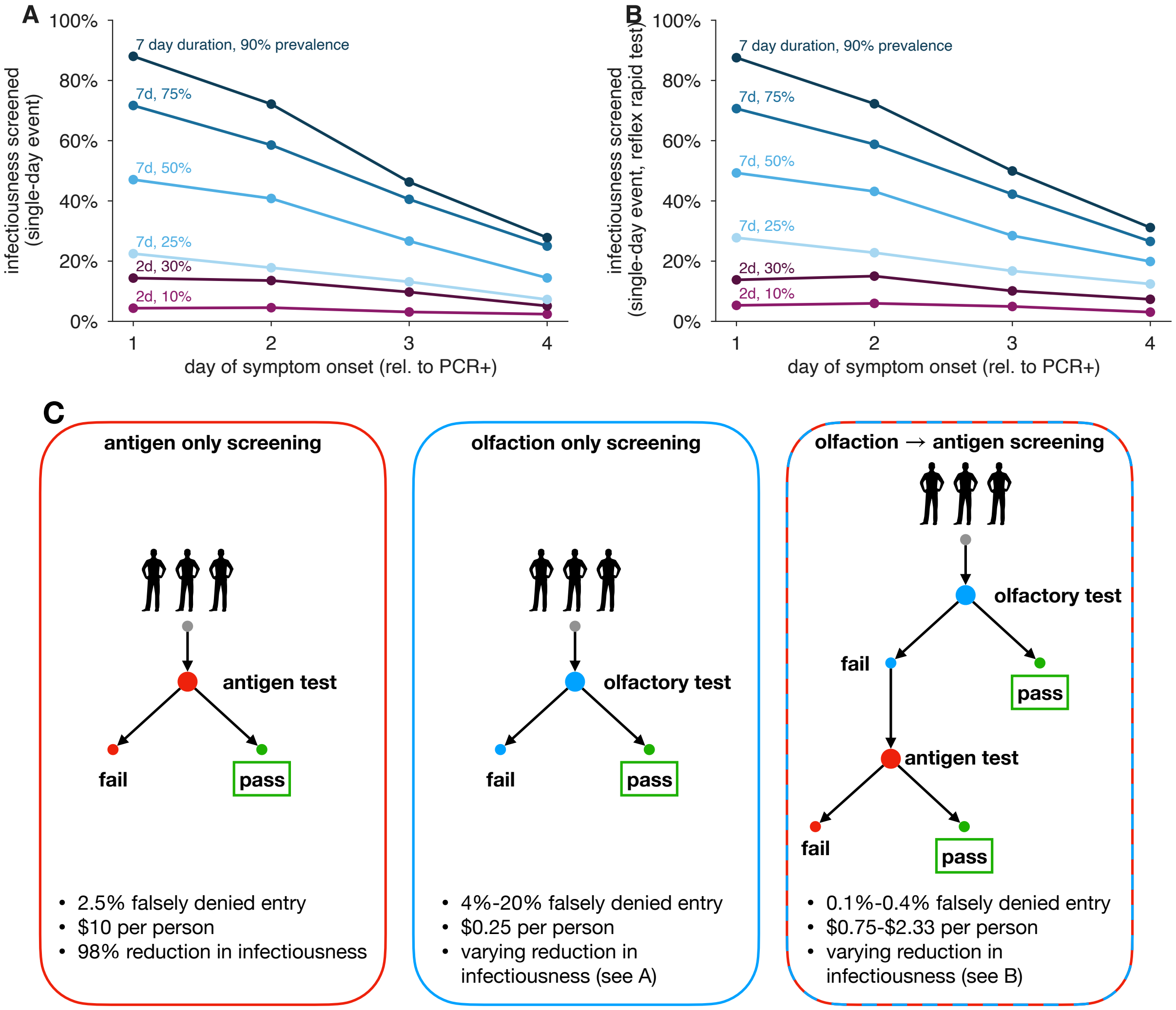

Supplementary Figure 5: **Olfactory dysfunction and antigen testing for point-of-entry screening.** (A,B) Lines show infectiousness screened for various combinations of symptom prevalence, duration, and onset, without (A) and with (B) reflex testing of those who fail the olfactory test with a rapid antigen test. Blue lines model olfactory dysfunction: 90% (dark blue), 75% (medium blue), 50% (light blue). Red lines model fever: 30% (dark red), 10% (light red). d, duration of symptom in days. (C) Diagram shows the impacts of antigen-based, olfaction-based, and olfaction+antigen screening as point of entry testing. The combined olfaction+antigen approach results in a low-cost, highly effective, and high specificity point-of-entry screening. d, days.

Supplementary Note

1 Predicting the impact of repeated population screening testing on R

The impact of repeated population screening on the reproductive number can be estimated by considering the ratio of population infectiousness with a screening regimen to population infectiousness with no screening. However, note that the impact of a population screening policy may depend on two additional factors.

First, not all individuals may wish to participate in a testing program. Let the fraction of individuals who participate be given by ϕ.

Second, a virological test (e.g. RT-PCR) may produce a false negative result unrelated to its limit of detection—for instance due to an improperly collected sample. Let se be the test sensitivity, in the particular sense of the probability of correctly diagnosing an individual as positive when that person's viral load should, in principle, have provided a sufficiently high RNA concentration to be detectable.

Let f_0 be the total infectiousness removed with no testing policy, i.e. due to symptom-driven self isolation. Let $f_{\text{test}}(se)$ be the fraction of total infectiousness removed with a chosen testing policy, inclusive of symptom-driven self isolation, as well as the test sensitivity se introduced above. The quantity f_{test} may be computed for any screening program, including virological testing or symptom screening.

Both f_0 and $f_{\text{test}}(se)$ can be estimated rapidly via Monte Carlo by drawing trajectories and applying a population screening regimen to them in which a fraction $1 - se$ positive tests are discarded uniformly at random. In the main text, we found that estimating these values using $10,000$ randomly drawn trajectories was sufficient to produce stable estimates.

Under the assumption of statistical independence between an individual's participation or refusal, viral load or olfactory dysfunction status, and se, we can approximate the reproductive number as

$$R \approx \left[\phi \frac{1 - f_{\text{test}}(se)}{1 - f_0} + 1 - \phi\right] R_0 \;, \tag{1}$$

which simply expresses a weighted combination of removed infectiousness via screening regimen participation and no test. Intuitively, note that if there is complete refusal to participate ($\phi = 0$) or an entirely ineffective test ($f_{\text{test}}(se) = f_0$), then $R \approx R_0$, as expected.

References

[1] Shima T Moein, Seyed MR Hashemian, Babak Mansourafshar, Ali Khorram-Tousi, Payam Tabarsi, and Richard L Doty. Smell dysfunction: a biomarker for COVID-19. In *International forum of allergy & rhinology*. Wiley Online Library, 2020.

[2] Luigi Angelo Vaira, Giovanni Salzano, Marzia Petrocelli, Giovanna Deiana, Francesco Antonio Salzano, and Giacomo De Riu. Validation of a self-administered olfactory and gustatory test for the remotely evaluation of COVID-19 patients in home quarantine. *Head & neck*, 42(7):1570–1576, 2020.

[3] Daniel Hornuss, Berit Lange, Nils Schroeter, Siegbert Rieg, Winfried V Kern, and Dirk Wagner. Anosmia in COVID-19 patients. *Clinical Microbiology and Infection*, 2020.

[4] Jerome R Lechien, Carlos M Chiesa-Estomba, Daniele R De Siati, Mihaela Horoi, Serge D Le Bon, Alexandra Rodriguez, Didier Dequanter, Serge Blecic, Fahd El Afia, Lea Distinguin, et al. Olfactory and gustatory dysfunctions as a clinical presentation of mild-to-moderate forms of the coronavirus disease (COVID-19): a multicenter european study. *European Archives of Oto-Rhino-Laryngology*, pages 1–11, 2020.

[5] Luigi Angelo Vaira, Giovanna Deiana, Alessandro Giuseppe Fois, Pietro Pirina, Giordano Madeddu, Andrea De Vito, Sergio Babudieri, Marzia Petrocelli, Antonello Serra, Francesco Bussu, et al. Objective evaluation of anosmia and ageusia in COVID-19 patients: Single-center experience on 72 cases. *Head & neck*, 42(6):1252–1258, 2020.

[6] Luigi Angelo Vaira, Claire Hopkins, Giovanni Salzano, Marzia Petrocelli, Andrea Melis, Marco Cucurullo, Mario Ferrari, Laura Gagliardini, Carlotta Pipolo, Giovanna Deiana, et al. Olfactory and gustatory function impairment in COVID-19 patients: Italian objective multicenter-study. *Head & neck*, 42(7):1560–1569, 2020.

[7] Anindya S Bhattacharjee, Samir V Joshi, Shilpa Naik, Shashikala Sangle, and Nixon M Abraham. Quantitative assessment of olfactory dysfunction accurately detects asymptomatic COVID-19 carriers. *EClinicalMedicine*, page 100575, 2020.

[8] Yury Rusetsky, Irina Meytel, Zhanna Mokoyan, Andrey Fisenko, Anna Babayan, and Ulyana Malyavina. Smell status in children infected with SARS-CoV-2. *The Laryngoscope*, 2021.

[9] Akosua Adom Agyeman, Ken Lee Chin, Cornelia B Landersdorfer, Danny Liew, and Richard Ofori-Asenso. Smell and taste dysfunction in patients with COVID-19: A systematic review and meta-analysis. In *Mayo Clinic Proceedings*. Elsevier, 2020.

[10] Mackenzie E Hannum, Vicente A Ramirez, Sarah J Lipson, Riley D Herriman, Aurora K Toskala, Cailu Lin, Paule V Joseph, and Danielle R Reed. Objective sensory testing methods reveal a higher prevalence of olfactory loss in COVID-19 –positive patients compared to subjective methods: A systematic review and meta-analysis. *Chemical senses*, 45(9):865–874, 2020.
